# A Theoretical Study on Seasonality

**DOI:** 10.3389/fneur.2015.00094

**Published:** 2015-05-07

**Authors:** Christoph Schmal, Jihwan Myung, Hanspeter Herzel, Grigory Bordyugov

**Affiliations:** ^1^Institute for Theoretical Biology, Charité Universitätsmedizin, Berlin, Germany; ^2^RIKEN Brain Science Institute, Wako, Japan; ^3^Institute for Theoretical Biology, Humboldt Universität zu Berlin, Berlin, Germany

**Keywords:** circadian clock, entrainment, seasonality, oscillator

## Abstract

In addition to being endogenous, a circadian system must be able to communicate with the outside world and align its rhythmicity to the environment. As a result of such alignment, external Zeitgebers can entrain the circadian system. Entrainment expresses itself in coinciding periods of the circadian oscillator and the Zeitgeber and a stationary phase difference between them. The range of period mismatches between the circadian system and the Zeitgeber that Zeitgeber can overcome to entrain the oscillator is called an entrainment range. The width of the entrainment range usually increases with increasing Zeitgeber strength, resulting in a wedge-like Arnold tongue. This classical view of entrainment does not account for the effects of photoperiod on entrainment. Zeitgebers with extremely small or large photoperiods are intuitively closer to constant environments than equinoctial Zeitgebers and hence are expected to produce a narrower entrainment range. In this paper, we present theoretical results on entrainment under different photoperiods. We find that in the photoperiod-detuning parameter plane, the entrainment zone is shaped in the form of a skewed onion. The bottom and upper points of the onion are given by the free-running periods in DD and LL, respectively. The widest entrainment range is found near photoperiods of 50%. Within the onion, we calculated the entrainment phase that varies over a range of 12 h. The results of our theoretical study explain the experimentally observed behavior of the entrainment phase in dependence on the photoperiod.

## 1 Introduction

### 1.1 Entrainment

Most living organisms possess an internal clock which enables them to account for the periodically changing environment due to the Earth’s rotation. The clock has to be sufficiently precise and, which is sometimes more important, synchronized to the external cues referred to as Zeitgebers. The process of setting the internal clock by Zeitgebers is called entrainment. Light, being one of the strongest Zeitgebers, succumbs to seasonal changes which results in seasonal variations of the light–dark (LD) ratio. The present paper is a systematic investigation of how entrainment of circadian oscillators is influenced by seasonal variations of the Zeitgeber.

### 1.2 Seasonality

The first studies of the circadian seasonality go back nearly 50 years ago, see e.g., Ref. (1). The LD ratio has been identified as one of the factors that influence the phase of circadian entrainment. In agreement with intuition, the LD ratio close to 12 h:12 h was found to be the strongest Zeitgeber (2). In the golden hamster, the phase of entrainment was measured in dependence on the Zeitgeber period *T* for different LD ratios (3), suggesting that phase of entrainment is more sensitive to variations of *T* for shorter LD values. Latitude-dependent LD effects in *Drosophila auraria* were reported in Ref. (4), including differences in phase-response curves and the dependence of the entrainment phase on the photoperiod. In fruit flies, it has been found that the morning and evening activity is controlled by two distinctive sets of neurons (5), which support the idea of morning and evening oscillators (6).

More recently, an in-depth study of entrainment of *Neurospora crassa* under different photoperiods resulted in a three-parameter “circadian surface” (7). Using three different strains with free-running periods of τ = 16.5 h, τ = 22.5 h, and τ = 29 h, the phase of entrainment was measured for Zeitgebers of different LD ratios. On the molecular level, differences between responses to varying photoperiods were recently documented (8). The SCN - the central circadian pacemaker in mammals - showed a break up of synchronization under long photoperiods, but synchronization was re-attained after the transition to short days by advancing the decline of the expression of clock genes (8).

Those studies of seasonality were paralleled by computational modeling of circadian systems under Zeitgebers of different amplitudes, periods, LD ratios, and the proportion of the twilight within a day (9). Numerical calculations revealed that the LD ratio shifts the phase of entrainment in a direction which depends on whether the organism is day- or night-active. Another theoretical approach to the studies of phase of entrainment under different photoperiods was to use a simple piece-wise linear PRC model in Ref. (6). The main result was that the activity onset (dusk for night-active and dawn for day-active organisms) can be conserved across a variety of photoperiod conditions. In addition, a large body of the behavior of entrainment phase for different photoperiods and PRC types was produced and thoroughly analyzed (6).

### 1.3 Arnold tongue

**Figure 1:**
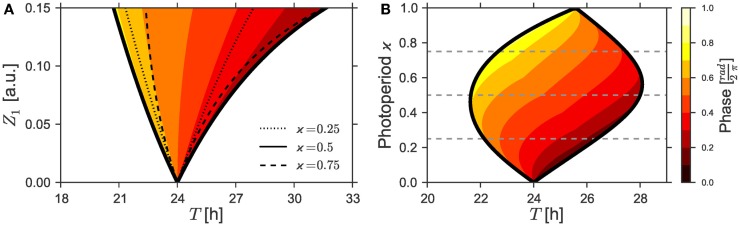
**(A)** 1:1 synchronization region in the *Z*_1_ − *T* parameter plane (1:1 Arnold tongue). *Dotted*, *bold*, and *dashed black* lines denote bifurcation curves of periodic solutions as determined by a continuation method for different photoperiods ϰ = 0.25, ϰ = 0.5, and ϰ = 0.75. Phases of the solutions in the 1:1 synchronization regime to equinox photoperiods (i.e., ϰ = 0.5) are color-coded. **(B)** 1:1 Entrainment range (Arnold onions) and color-coded phases in the ϰ–*T*-plane given a Zeitgeber strength of *Z*_1_ = 0.1. *Dashed lines* denote the corresponding photoperiods ϰ = 0.25, ϰ = 0.5, and ϰ = 0.75. Simulations for both Figures relied on the parameters τ = 24 h, λ = 0.5 h^−1^, and *A* = 1. The color-coded phases in the entrainment regions were determined by a “brute force integration method” as described in Section 2.3.

Graphically, entrainment is represented by the Arnold tongue - a triangular region on the “Zeitgeber period - Zeitgeber strength” parameter plane, see Ref. (10–12), compare also Figure 1A. Within the tongue, Zeitgeber enforces its period in the circadian system. When entrained, the phase of the circadian system assumes a stable relation to the phase of the Zeitgeber in such a way that *ψ* - the difference between those two phases - assumes a stable value. The range of period detunings between the Zeitgeber and the circadian system where entrainment occurs is called range of entrainment. Larger Zeitgeber strengths often lead to larger entrainment ranges which results in the characteristic wedge-like shape of the tongue. Within the Arnold tongue, the structure of the isophases has been determined (13), thus making possible to understand how the phase of entrainment changes under variations of Zeitgeber strength and period.

The classical Arnold tongue depicts the entrainment region in dependence on the effective Zeitgeber strength. When considering entrainment by Zeitgebers with different LD ratios, it is often unclear what the effective Zeitgeber strength is under varying LD ratio. Intuitively, one can expect that Zeitgebers with very short or very long light phase should be less potent in entraining the circadian system than a Zeitgeber with a LD ratio close to 12 h:12 h. In this manuscript, we quantify this intuition and compare it to previously published data on entrainment under different seasonal conditions (3, 14, 15).

### 1.4 Arnold onion

Our main result is the existence of the onion-shaped entrainment zone on the photoperiod-detuning parameter plane, compare Figure 1B. Both tips of the entrainment onion point to free-running periods in complete darkness τ_DD_ or constant light τ_LL_. The widest part of the entrainment range is close to the equinoctial photoperiod. The onion entrainment region is skewed to the right or to the left depending on whether τ_LL_ is larger or smaller than τ_DD_. Within the entrainment onion, we calculated the phases of entrainment, thus quantifying the claim by Aschoff (1) on the dependence of the entrainment phase on the photoperiod. The skewness of the entrainment onion makes possible achieving all possible phases of entrainment by changing photoperiods. Even for a constant mismatch τ − *T*, i.e., for vertical cross-sections of the Arnold onion, large variations of entrainment phases are found.

## 2 Materials and Methods

### 2.1 Mathematical model

In this paper, we use as an illustrative, conceptual model of the circadian clock, the generic amplitude–phase-oscillator

(1) $$\begin{array}{ccc} \frac{\text{d}r\left(t\right)}{\text{d}t} & =\lambda \;r\left(t\right)\;\left(A-r\left(t\right)\right), & \\ \frac{\text{d}\varphi \left(t\right)}{\text{dt}} & =2\;\pi \left[\varepsilon \;\cos^{2}\left(\varphi \left(t\right)∕2\right)+c\left(\varepsilon ,\tau \right)\right]\;, \end{array}$$

given in polar coordinates. Here, *r*(*t*) is the radial component while *φ*(*t*) describes the phase evolution. The model depends on a small set of generic parameters, namely the oscillator amplitude *A*, the amplitude relaxation rate λ, and the intrinsic period τ. The parameter ε controls the phase velocity $\frac{\text{d}\varphi \left(t\right)}{\text{d}t}$: for ε = 0 h^−1^, the angular velocity $\frac{\text{d}\varphi \left(t\right)}{\text{d}t}$ is constant along the whole limit cycle, and for ε ≠ 0 h^−1^, the limit cycle has sections of faster and slower changing $\frac{\text{d}\varphi \left(t\right)}{\text{d}t}$ (16).

The internal period τ of system (Eq. 1) can be defined by means of the time required for *φ*(*t*) to change by 2π, i.e.,

(2) $$\tau :=2\overset{\pi }{\underset{0}{\int }}\;\frac{\text{d}\varphi }{\dot{\varphi }\left(t\right)}=\frac{1}{\sqrt{c\left(c+\varepsilon \right)}}\;.$$

It can be noticed that τ generally depends on the choice of *c* and ε. For the sake of tunability, we have chosen the offset *c*(ε, τ) as

(3) $$c\left(\varepsilon ,\tau \right):=\sqrt{\tau^{-2}+\varepsilon^{2}∕4}-\varepsilon ∕2\;,$$

such that the internal period τ in system (Eq. 1) can be freely chosen for any given value of ε. If Eq. 1 adopts a uniform phase velocity $\dot{\varphi }$ = 2π∕τ for ε = 0, the model is commonly known as the *Poincaré oscillator* (11, 17, 18).

### 2.2 Zeitgeber input function

**Figure 2:**
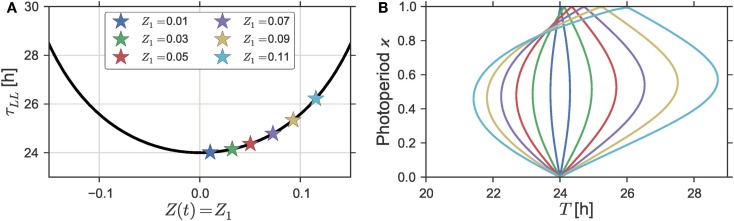
**(A)** Free-running period τ as a function of the intensity *Z*_1_ of a constant forcing signal, i.e., *Z*(*t*) = *Z*_1_ for all times *t*, which is equivalent to setting ϰ to one in Eq. 5. **(B)** Dependence of the entrainment region on varying intensities *Z*_1_ of a rhythmic Zeitgeber *Z*(*t*) as defined by Eq. 5 in Section S1.3 in Supplementary Material. A Zeitgeber steepness of *S* = 100 was used. Simulations were done for a (uniform) Poincaré oscillator with parameters ε = 0 h^−1^, *A* = 1, and λ = 0.5 h^−1^. A three-dimensional representation of **(B)** can be found in Figure S3 in Supplementary Material.

Equation 1 can be transferred into Cartesian coordinates using the definitions *x*(*t*): = *r*(*t*) cos(φ(*t*)) and *y*(*t*): = *r*(*t*) sin(φ(*t*)). Including an additive Zeitgeber term, Eq. 1 then reads

(4) $$\begin{array}{ccc} \frac{\text{d}x\left(t\right)}{\text{d}t} & =f_{x}\left(x\left(t\right),y\left(t\right)\right)+\cos\left(\alpha \right)\;Z\left(t\right), & \\ \frac{\text{d}y\left(t\right)}{\text{d}t} & =f_{y}\left(x\left(t\right),y\left(t\right)\right)+\sin\left(\alpha \right)\;Z\left(t\right), \end{array}$$

where *Z*(*t*) is a scalar *T*-periodic Zeitgeber function. An explicit representation of the vector field $\vec{f}\left(\vec{x}\left(t\right)\right)\;:={\left(f_{x}\left(x\left(t\right),y\left(t\right)\right),f_{y}\left(x\left(t\right),y\left(t\right)\right)\right)}^{T}$ is given by Eq. 4 in Section S1.1 in Supplementary Material. Finally, α denotes the “direction” of the perturbation of amount *Z*(*t*) that is applied to the vector field $\vec{f}\left(\vec{x}\left(t\right)\right)$. We define this direction by means of the azimuth α in the polar plane. Using the relations cos(α + π) = −cos(α) and sin(α + π) = −sin(α), we can deduce that a phase-shift of π (or 180°) in the direction α of the perturbation is tantamount to substituting *Z*(*t*) by −Z(*t*). We will use this relation for the interpretation of Figure 2A as well as Figures S2 and S4A in Supplementary Material.

In analogy to common Zeitgeber signals under laboratory conditions, we investigate the entrainment of the *Poincaré Oscillator* to square-wave cycles. Such binary Zeitgeber can be written as

(5) $$Z_{⊓}\left(t\right):=\left\{\begin{array}{cc} Z_{1} & \;\forall t:\;t\;\text{mod}\left(T\right)\leq \mathrm{\varkappa T}\\ 0 & \;\text{elsewhere} \end{array}\right.$$

where we define the photoperiod $\varkappa :=\frac{T_{Z_{1}}}{T}\in \left[0,1\right]$ as the duration of the phase $T_{Z_{1}}$ with an active Zeitgeber signal (i.e., *Z*(*t*) = *Z*_1_) divided by the period *T* of one Zeitgeber cycle. The photoperiod parameter ϰ thus represents the relative duration of the light phase during the day, i.e.,

$$\varkappa =\frac{\text{LL}}{\text{LL}+\text{DD}}.$$

Equation 5 inevitably leads to discontinuities of Z_⊓_(*t*) at all time points of changing Zeitgeber intensity. For the sake of the numerical stability of continuation methods applied below, we substitute the piece-wise linear Zeitgeber function (Eq. 5) by a continuous approximation *Z*(*t*), details are given in Section S1.3 in Supplementary Material.

In Section 3.5, we substitute the entrainment signal *Z*(*t*) by the Fourier expansion of the asymmetric square-wave signal Z_⊓_(*t*). The partial sum including the first *N* summands of the Fourier series *ℱ*{*Z*(*t*)} can be written as

(6) $$Z_{N}\left(t\right)=Z_{1}\;\varkappa +\sum_{k=1}^{N}\;R_{k}\text{ cos}\left(2\;\pi \;k\;t\;∕\;T+\phi \right)\;,$$

using the abbreviations ϕ:=arctan(−*b_k_*/*a_k_*) and $R_{k}:=\sqrt{a_{k}^{2}+b_{k}^{2}}$ with the Fourier coefficients $a_{k}=\frac{Z_{1}}{k\;\pi }\sin\left(2\;\pi \;k\;\varkappa \right)$ and $b_{k}=\frac{Z_{1}}{k\;\pi }\left(1-\cos\left(2\;\pi \;k\;\varkappa \right)\right)$, see Section S1.4 in Supplementary Material for a derivation of Eq. 6.

### 2.3 Numerics

**Figure 3:**
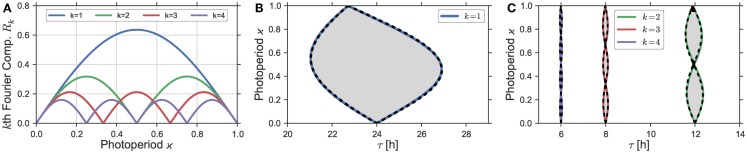
**(A)** Plotted are the Fourier coefficients *R_k_*(ϰ) of order *k* = 1, 2, 3, 4 from the Fourier decomposition (Eq. 6) of the asymmetric square-wave signal (Eq. 5). **(B,C)** Entrainment regions in the ϰ − *t* parameter plane. *Gray* areas denote these entrainment regions as determined by the “brute force integration method” as described in Section 2.3. *Dashed black lines* denote the entrainment border for system (Eq. 4), driven by the rectangular Zeitgeber signal *Z*(*t*) from Eq. 15 in Section S1.3 in Supplementary Material, using a Zeitgeber steepness of *S* = 100. *Bold colored lines* denote the borders of entrainment of the same system (Eq. 4) in case it is driven by the sum of the zeroth and *k*-th Fourier mode from the Fourier expansion (Eq. 6). Zeitgeber and oscillator properties are given by *Z*_1_ = 0.1, and *T* = 24 h as well as *A* = 1, λ = 0.5 h^−1^, and ε = 0 h^−1^, respectively.

The color-coded entrainment regions of Figures 1, 3B,C, and 5A as well as the phases in the Figures S4 and S5B in Supplementary Material were calculated by the following “brute force integration method”: firstly, after choosing all Zeitgeber and oscillator parameters, we integrated system (Eq. 4) in Cartesian coordinates for the period of 105 entrainment cycles *T* using the *SCIentificPYthon* function *odeint*. After that, we determined whether the oscillator is entrained to the Zeitgeber signal by the following procedure: We take the state ${\vec{x}}_{0}$ of the system at the beginning of the 85th entrainment cycle and determine the recurrence times *t_n_*, *n* = 0, …, *N*_max_, to state ${\vec{x}}_{0}$ for all times *t* > 85 *T*. We assume that the system has returned to the state ${\vec{x}}_{0}$, if it has entered a small neighborhood of ${\vec{x}}_{0}$ in state space, defined by an ϵ-ball $B_{\in }\left({\vec{x}}_{0}\right)\;:\;=\{\vec{x}\;\in {\mathbb{R}}^{\dim\left({\vec{x}}_{0}\right)}:\;{\left\|\vec{x}-{\vec{x}}_{0}\right\|}_{2}<\epsilon \}$ with ϵ = 0.01 (such that it can return at all, given the inaccuracies of numerical integration). If the recurrence times do not change over time (i.e., $|t_{n+1}-t_{n}|<\delta$ with δ being small) and, on top of that, constitute a rational multiple of the Zeitgeber period *T*, we consider the oscillator entrained. Finally, we define the phases of *x*(*t*) and *y*(*t*) of the entrained oscillator based on the time a given variable needs to reach a local maximum after the onset of light.

Apart from this “brute force integration method,” we also calculated the borders of entrainment, which are given by bifurcations of the system (Eq. 4). For this purpose, we used two different Continuation software packages: Figure 2A as well as Figures S2, S3, S4A, and S5A in Supplementary Material rely on computations done with *XPP-AUTO* (19). All other bifurcation lines in Figures 1, 2B, 3B,C as well as Figure 5A were computed by means of *AUTO-07p* (20, 21).

## 3 Results

Circadian clocks are endogenous pacemakers, generating oscillations of certain physiological processes with a period of approximately 24 h. One of the central properties of these rhythms is their persistence under constant environmental conditions, i.e., without fluctuating external influences at diel frequency. However, constant environmental conditions are rather the extreme exception in nature. Thus, to pace the rhythmic endogenous processes with the period of the Earth’s rotation is of major importance and was shown to confer a fitness benefit to the organism (22–24). Rhythmic environmental signals that are able to synchronize or entrain the circadian system to their own frequency are called *Zeitgeber* signals (25).

In case of synchronization, the circadian oscillation of a given physiological process and the Zeitgeber signal will establish a stable phase relation *ψ*, commonly known as the phase of entrainment (POE). If the system synchronizes at all and in such case the POE will generally be dependent on the intrinsic properties of the circadian system as well as the waveform, intensity, and period of the Zeitgeber signal.

In the next sections, we will systematically investigate the dependency of the entrainment region and the POE on the properties of the oscillator and the Zeitgeber signal by means of modeling approaches. We will show that entrainment under different photoperiod bears many similarities with the classical Arnold tongue picture. The calculated dependence of the POE on the photoperiod parameter ϰ and the oscillator’s intrinsic period τ will complete our study.

### 3.1 Mathematical model

Over the last decades, mathematical models have made a decisive contribution to the understanding of the circadian clockwork among a variety of organisms. While detailed, biochemically motivated models aim to shed light on the interlocking of the circadian clockwork’s cogs and levers in a specific biological context (organisms, cell types, etc.), simple and rather abstract conceptual oscillator models can be used to understand generic features of the circadian system (26).

Following this tradition of modeling in the field of chronobiology (9, 27–31), we describe the dynamical properties of the circadian clock by means of a generic amplitude–phase oscillator, see Section 2.1 for details. Given in polar coordinates, the dynamical Eq. 1 define the radial evolution *r*(*t*) in time (i.e., the time-dependent distance from the origin) as well as the phase dynamics *φ*(*t*) for a given initial condition (*r*_0_, *φ*_0_). A small set of only four generic parameters τ, *A*, λ, and ε conveniently describes general features of limit cycle oscillators: While *A* denotes the amplitude of the oscillator with internal period τ, the parameter λ quantifies the rate at which an amplitude perturbation relaxes back to its stable periodic orbit at radius r^⋆^ = A. Parameter ε determines the shape of the oscillations. For ε = 0 h^−1^, Eq. 1 reduce to a system of uniform phase velocity which is commonly referred to as a *Poincaré oscillator* (17). For ε > 0 h^−1^, we introduce a non-uniformity of the phase velocity: on the periodic orbit, there are stretches of a larger and smaller instantaneous phase velocity $\frac{\text{d}\varphi \left(t\right)}{\text{d}t}$. We can thus tune the shape of the resulting oscillations (when viewed in Cartesian coordinates) ranging from sinusoidal (ε = 0 h^−1^) to more and more spike-like oscillations (ε > 0 h^−1^), compare also Ref. (16).

### 3.2 Entrainment

Throughout this paper, we consider the effect of a given Zeitgeber by means of a scalar function *Z*(*t*) that forces the system along a certain direction (e.g., the *x*- or *y*-axis) in the phase-plane, see Eq. 4. Recently, it was demonstrated that oscillators with low amplitudes *A* and relaxation rates λ can be more easily entrained by a rhythmic Zeitgeber *Z*(*t*) in comparison to oscillators with large *A* and λ (32). Based on this differential responsiveness to a certain Zeitgeber signal, we will term oscillators with a large entrainment region “weak” oscillators while terming oscillators with a narrow entrainment region “strong” oscillators.

As already discussed above, the strength, period, and waveform of a Zeitgeber signal will determine if an oscillator will entrain or not. Figure 1A shows the region of 1:1 synchronization in the parameter plane of Zeitgeber intensity *Z*_1_ and period *T*. A square-wave Zeitgeber signal with equal periods of light and darkness was assumed to act on a *uniform Poincaré oscillator* with ε = 0 h^−1^, *A* = 1, λ = 0.5 h^−1^, and an intrinsic period of τ = 24 h. The phases of entrainment, which were normalized to values between 0 and 1, are color-coded. As intuitively expected, narrow entrainment ranges can be observed for a Zeitgeber signal of low strength *Z*_1_. The entrainment range successively gets broader with an increasing Zeitgeber intensity *Z*_1_, thus leading to a triangularly shaped structure called Arnold tongue. The tip of this Arnold tongue lies for *Z*_1_ = 0 at a point defined by a vanishing period mismatch (i.e., τ − *T* = 0).

Since the effective Zeitgeber strength scales reciprocally with the oscillator amplitude (13), we can interpret the effect of a low or high Zeitgeber intensity *Z*_1_ in analogy to the behavior of a strong or a weak oscillator, respectively. Thus, it follows from Figure 1A that strong oscillators with a small range of entrainment exhibit a high sensitivity of their phases of entrainment to the period mismatch τ − *T*. Analogously, weak oscillators with a large range of entrainment exhibit a low sensitivity of their phase of entrainment.

The discrimination of weak and strong oscillators was used in Ref. (32) to interpret experiments on the tissue level but can even be applied to interpret entrainment data on the organismic level. For example, a comparative study on literature data by Aschoff and Pohl revealed that mammals and birds have a rather narrow entrainment range accompanied by a high sensitivity of the entrainment phase *ψ* in comparison to insects, plants, and unicellular organisms (3). These differences could either reflect a differential response to a given Zeitgeber signal or could point to different oscillator properties of the underlying circadian clocks.

An attractive hypothesis to explain such differences in entrainment ranges and phase sensitivities relies on the effect of mutual coupling among clock neurons in the mammalian and avian circadian system. In contrast to unicellular organisms and plants, vertebrates have a highly centralized organization of their circadian master clock, consisting of multiple, densely packed neurons which are thought to mutually couple via neurotransmitters like GABA, VIP, or AVP (33). However, it was shown theoretically that coupling between autonomously oscillating systems can lead to an amplitude expansion as well as an enhancement of the relaxation rates of the oscillators. It can thus reduce the entrainment range of the coupled system in comparison to the behavior of the single oscillators (32, 34). Along these lines, coupling between clock neurons could be the essential difference that explains the narrower entrainment range and higher sensitivity of *ψ* with respect to varying period mismatches τ − *T* in mammals and birds. It shall be noted, that the above interpretation for ensembles of cells, which is supported e.g., by entrainment data in Ref. (3, 32), solely holds true where the dynamical behavior of the whole network of coupled oscillators can be approximated by a single-oscillator model. The interpretation of certain other experimental findings might demand the consideration of the underlying network organization: one example is an enhanced sensitivity to Zeitgeber signals during an increasing ensemble amplitude of neuronal activity in a population of clock neurons after short-day entrainment in mice (35). A similar boost of phase-shift capacity after short-day entrainment was found in hamsters (36). These findings, at a first glance counterintuitive in the light of the differences between strong and weak oscillators, could indicate yet to be clarified network mechanisms.

### 3.3 Effects of a varying photoperiod

So far, we have discussed the impact of varying Zeitgeber intensity and period mismatch on the entrainment properties of the circadian clock. Organisms living distant from equatorial latitudes are also subject to seasonal changes in the duration of light they receive per day. We now investigate, using our modeling approach, the effect of varying light duration by changing the fraction ϰ of one Zeitgeber period *T* in which the Zeitgeber signal is active (i.e., *Z*(*t*)=*Z*_1_), see Section 2.2 and Section S1.3 in Supplementary Material for technical details. Throughout the rest of this paper, such fraction ϰ, which is also termed *duty cycle* in the context of electrical engineering, will be used as synonym for the *photoperiod*. The photoperiod ϰ can take values between 0 and 1 and the extremal values ϰ = 0 and ϰ = 1 are equivalent to constant darkness or constant light conditions, respectively.

Figure 1B investigates the region of entrainment in the ϰ − *T* parameter plane for a maximal Zeitgeber intensity of *Z*_1_ = 0.1, using the same set of oscillator parameters as in Figure 1A (i.e., ε = 0 h^−1^, *A* = 1, λ = 0.5 h^−1^, and τ = 24 h). In the first place, one notices that the entrainment region adopts an oval, onion-shaped geometry. Such region that we will term Arnold onion in the following, has its widest range of entrainment near the equinoctial photoperiods at ϰ = 0.5. It gets narrower for photoperiods differing from equinox and tapers toward extremer photoperiods. The tips of the onion point to entrainment periods *T* that are given by the free-running periods of the oscillator under constant darkness (τ_DD_) or constant light (τ_LL_), respectively. Thus, the Arnold onion will be tilted (i.e., there is no symmetry along the ϰ-axis) in any case where the difference Δτ = τ_LL_ − τ_DD_ does not equal 0.

The Arnold onion represents the main finding of this paper. We continue the manuscript by explaining the following aspects: (i) what determines the tilt of the onion, i.e., whether the onion is skewed toward left or toward right, (ii) what makes the Arnold onion open and close again under change of the photoperiod and how this can be related to the properties of the classical Arnold tongue, and (iii) what is the distribution of the phase of entrainment *ψ* within the onion.

### 3.4 Free-running periods determine the tilt of the arnold onion

The influence of a certain Zeitgeber signal can be interpreted as a parametric change in the circadian clocks dynamical system, i.e., a time-dependent change of its set of parameters. Changes in the intensity of a constant environmental signal that can potentially act as a Zeitgeber will thus most probably lead to changes in the oscillation period whenever its parametric effects are not compensated or balanced out. A large body of data has accumulated, showing for a variety of organisms that the free-running period under conditions of a constant light generally depends on the intensity of the illumination (37, 38). Whether such parametric changes lead to an increase or decrease of the free-running period for an increasing light-intensity generally depends on the specificities of the organism under investigation. Aschoff was the first who noticed that day-active animals and green plants typically shorten (τ_LL_ < τ_DD_) while night-active animals lengthen (τ_LL_ > τ_DD_) their free-running period with an increasing intensity of illumination (37). Only few exceptions have been found to this rule which is now known as *Aschoff’s Rule* (39). According to this rule, we expect that the Arnold onions of night-active animals like mice will be tilted to periods larger than their spontaneous frequency τ_DD_ under constant darkness. Analogously, Arnold onions of day-active animals and plants are expected to be tilted the other way around.

Since the tips of the Arnold onion are given by the free-running periods τ_DD_ (the lower tip) and τ_LL_ (upper tip), the tilt of the onion depends on the relation between those free-running periods. Figure 2A shows the dependency of the free-running period τ_LL_ of a uniform Poincaré Oscillator (i.e., ε = 0 h^−1^) to changing intensities of a constant forcing signal *Z*(*t*) = *Z*_1_ for all times *t*. The effect of a forcing signal is defined as an additive perturbation along the *x*-axis like before [i.e., α = 0 in Eq. 4. If we take *Z*_1_ = 0 as the nominal parameter value under constant darkness, a steady increase of the free-running period can be observed for steadily increasing Zeitgeber intensities with *Z*_1_ > 0. The only exception is a small range of high Zeitgeber intensities *Z*_1_ ≫ 0 close before a further increase would lead to arrhythmia (damped oscillations) by driving the system through an *Andronov*–*Hopf bifurcation*. Therein, we can observe a decline of the free-running period with an increasing Zeitgeber intensity, see Figure S2 in Supplementary Material. In general, the steepness of such light-intensity-dependent changes of the free-running period is strongly dependent of the oscillator properties. Uniform Poincaré oscillators with higher radial relaxation rates λ or lower amplitudes *A* exhibit a steeper increase in τ_LL_ upon changing *Z*_1_ compared to oscillators with small values of λ and high values of *A*, respectively, compare Figures S2A,B in Supplementary Material.

The effects of the maximal Zeitgeber intensity *Z*_1_ on the shape of the Arnold onion are investigated in Figure 2B. There, the entrainment regions in the ϰ − *T* parameter plane are plotted for different Zeitgeber intensities *Z*_1_. As expected from the dependency of the free-running period τ_LL_ on the Zeitgeber intensity *Z*_1_, see Figure 2A, we can observe an increasing tilt of the Arnold onion for increasing values of *Z*_1_. Since the width of the entrainment range of a self-sustained oscillator is usually positively correlated with the amplitude of its entraining Zeitgeber signal (see, e.g., Figure 1A), we see, apart from a larger tilt also a broadening of the Arnold onion with increasing Zeitgeber strength.

A peculiarity of the Poincaré Oscillator with ε = 0 h^−1^ is the uniform evolution of the oscillators’ phase in time. As a direct consequence, the system yields rotation equivariance. Thus, a rotation of the Zeitgeber direction α by a certain angle β will lead to exactly the same solution apart from a phase-shift of magnitude β, see Section S1.2 in Supplementary Material for further details. This explains the symmetry in Figure 2A, namely the fact that both positive and negative constant forcing signals result in increasing τ_LL_. As described in Section 2.2, negative values of *Z*_1_ correspond to a rotation of the Zeitgeber by β = 180°, thus leading to phase-shifted solutions with exactly the same period τ_LL_ compared to positive signals *Z*(*t*) (corresponding to α = 0°) of a given intensity |*Z*_1_|. Regardless of the direction of the forcing signal, we thus obtain an increase in the free-running period τ_LL_ with increasing Zeitgeber strength, which mimics the behavior of night-active animals.

In a simple extension of the model, the rotational symmetry of the uniform Poincaré Oscillator can readily be broken by introducing a non-uniform phase evolution as described by Eq. 1 for ε ≠ 0 h^−1^. Such non-uniform oscillator shows a decrease of the free-running period for *Z*_1_ < 0 and an increase for *Z*_1_ > 0 if a constant forcing signal is applied, see Figure S4A in Supplementary Material. Hence, the corresponding Arnold onions are tilted toward τ_LL_ < τ_DD_ for *Z*_1_ < 0 and into the opposite direction τ_LL_ > τ_DD_ for *Z*_1_ > 0, see Figure S4B in Supplementary Material. We are thus able to mimic this general behavior of night-active animals and day-active animals or plants with one generic oscillator model, parameterized by a set of only four generic parameters.

### 3.5 Explaining the form of the arnold onion

It turns out that the onion-like structure of the entrainment zone on the “LD ratio-detuning” parameter plane can be explained with the help of the reduction of the system to its phase dynamics (12). This explanation does not rely on a particular kind of the oscillating system, which we assume to have a most general form of

(7) $$\frac{\text{d}\vec{u}}{\text{d}t}=\vec{f}\left(\vec{u}\right)+\vec{z}\left(t\right),$$

where $\vec{u}(t)$ is a *N*-dimensional state variable, $\frac{\text{d}\vec{u}}{\text{d}t}=\vec{f}\left(\vec{u}\right)$ is the autonomous (non-perturbed) equation of the circadian oscillator model and $\vec{z}(t)$ is an external time-periodic Zeitgeber. It turns out (12, 13) that the dynamics of the phase difference *ψ* between the oscillations of $\vec{u}(t)$ and the Zeitgeber $\vec{z}(t)$ is determined by the circular convolution integral

(8) $$\frac{\text{d}\psi }{\text{d}t}=\overset{T}{\underset{0}{\int }}\text{ d}t\;\vec{\xi }\left(t-\psi \right)\vec{z}\left(t\right),$$

where $\vec{\xi }(t)$ is the infinitesimal phase-response function of the limit cycle in the unperturbed equation $\frac{\text{d}\vec{u}}{\text{d}t}=\vec{f}\left(\vec{u}\right)$. This convolution integral can be best understood by recalling that a convolution in the time domain is equivalent to the product of the Fourier transforms of the functions $\vec{\xi }$ and $\vec{z}$ in the frequency domain.

#### 3.5.1 The Case of Sine-Like PRCs

In the case of Eq. 1 with ε = 0 h^−1^, the phase-response function is given by $\vec{\xi }\left(t\right)={\left(-\sin\left(\omega t\right),\cos\left(\omega t\right)\right)}^{T}$, i.e., it has a single Fourier component at the base frequency ω. By the convolution theorem, the dynamics of the phase difference will hence be determined only by the first Fourier coefficient of the Zeitgeber *Z*(*t*). In Figure 3, we present the result of the computation of the Arnold onion in a direct way compared to the prediction by the first Fourier mode. Figure 3A shows the dependence of the first four Fourier modes of a rectangular Zeitgeber in dependence on the photoperiod ϰ, note also Eq. 6. There, we see that the first Fourier mode has its maximum at ϰ = 0.5 and approaches 0 for ϰ close to 0 and 1. Thus, we expect that Zeitgebers with ϰ close to 0.5 would have the largest influence on the circadian oscillator and, consequently, would result in a widest range of entrainment. In Figure 3B, we plot the Arnold onion calculated with the rectangular Zeitgeber with different photoperiods ϰ (bold colored lines) and, for comparison, the Arnold onion calculated using just the sum of zeroth and the first Fourier mode of the same Zeitgeber (dashed black lines). Both lines coincide almost perfectly, which supports our claim that the first Fourier mode of the Zeitgeber determines the entrainment dynamics in Eq. 1 with ε = 0 h^−1^.

#### 3.5.2 Higher Order Arnold Onions

So far, we have considered 1:1 entrainment in case of Zeitgeber periods *T* that were close to the internal period τ. The same logic applies for entrainment by Zeitgebers with periods *T* being multiples of the endogenous period τ with the only difference that it is the *k*-th Fourier mode that matters for entrainment with $\frac{T}{\tau }$ ≈ k for *k* = 1, 2, …. Depending on the photoperiod ϰ, the *k*-th Fourier mode of the rectangular Zeitgeber has *k* + 1 zeros, compare Figure 3A. The corresponding entrainment zones are consequently organized as *k* separate Arnold onions piled on top of each other, see Figure 3C. The photoperiods ϰ of the zero-width entrainment range are given by the values of ϰ, where the corresponding *k*-th Fourier mode has a 0. Higher order resonances have also been observed experimentally in the context of circadian entrainment, where the phenomenon is commonly known as *frequency demultiplication* (7, 40, 41).

#### 3.5.3 Beyond Sine-Like PRCs

Even if the PRC of the oscillator contains more than one Fourier mode (as it is the case in Eq. 1 with ε ≠ 0 h^−1^), we can still apply the convolution theorem to Eq. 8. For a constant Zeitgeber, be it a 0 as in the case of a DD regime or a positive constant as in the case of a LL regime, all Fourier modes save the zeroth, are equal to 0, thus making no contribution to the dynamics of the phase difference *ψ* in Eq. 8. Thus, we expect that even for a general non-sine-like PRC and an arbitrary Zeitgeber with a seasonally driven periodicity, the entrainment zone must close in an onion-like manner at ϰ = 0 and ϰ = 1.

### 3.6 Entrainment phases under varying photoperiods

Possessing an intrinsic period τ that enables the synchronization to environmental Zeitgeber signals allows the circadian clock to pace physiological processes with daily environmental cycles of light and temperature. In the synchronized state, it is actually the phase of entrainment *ψ* that schedules diurnal physiological processes within the temporal structure of a solar day. From an evolutionary point of view, parameters that influence the value of *ψ* are thus expected to be open to evolutionary adjustment or natural selection. Our current theoretical considerations allow us to study the phase of entrainment *ψ* inside the Arnold onions, i.e., for different values of period mismatches and photoperiods.

**Figure 4:**
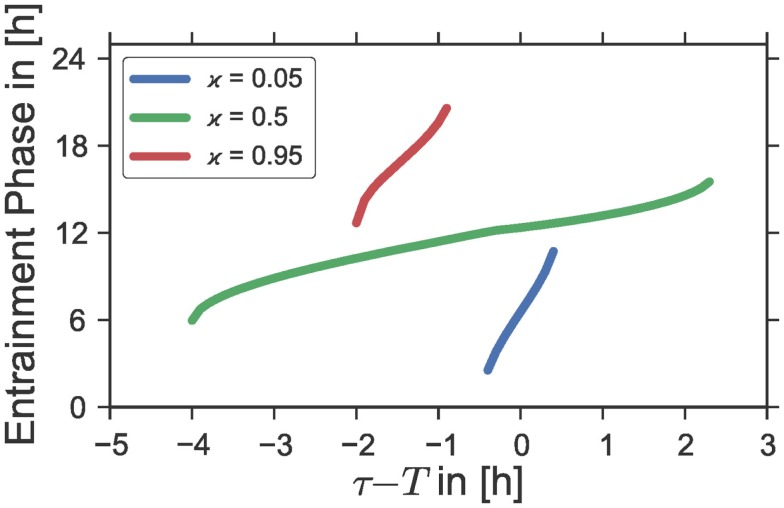
**Entrainment phases *ψ* as a function of the period mismatch τ − *T* between the intrinsic oscillator period τ and the Zeitgeber period *T* for different photoperiods ϰ**. The curves correspond to horizontal cross-section of Figure 1B at the ordinate positions ϰ = 0.05, ϰ = 0.5, and ϰ = 0.95, respectively; i.e., the same Zeitgeber intensity *Z*_1_ and oscillator properties ε, *A*, λ, and τ were used as those underlying the simulations in Figure 1B.

We have shown in Section 3.2 that the entrainment range of a given self-sustained oscillator which is subject to a periodic Zeitgeber signal broadens with an increasing Zeitgeber strength *Z*_1_. Interestingly, it is found that, regardless of the Zeitgeber strength, the phase of entrainment *ψ* can vary only within a range of approximately 180° across the entrainment interval of a classical Arnold tongue (13). Consequently, the sensitivity of the phase *ψ* to changes in the period mismatch τ − *T* gets smaller with increasing Zeitgeber intensities, see Figure 1A. Since we have proven in the last section that a rectangular Zeitgeber has its largest impact at equinoctial photoperiods (ϰ = 0.5), we expect the phase-sensitivity on the inside of a given Arnold onion to be smallest at ϰ = 0.5 while it should get larger the further we get away from equinox. This theoretical expectation is confirmed numerically by Figure 4, where we have plotted the phase of entrainment versus the period mismatch for equinoctial (ϰ = 0.5) as well as extremely short (ϰ = 0.05) and long (ϰ = 0.95) photoperiods. Interestingly, such behavior can also be found in experimental studies using golden hamsters (*Mesocricetus auratus*) (3). When forced by light–dark cycles of different periods *T*, these animals show a relatively wide range of entrainment and a low phase-sensitivity for equinoctial photoperiods ϰ = 0.5. In contrast, a high phase-sensitivity accompanied by a narrow range of entrainment could be observed for extremely short photoperiods where a 1 h light-pulse per Zeitgeber period *T* was applied.

**Figure 5:**
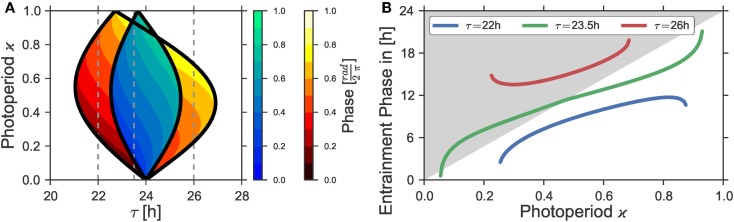
**(A)** Entrainment regions and color-coded entrainment phases in the ϰ − *t* parameter plane are plotted for *T* = 24 h and two different Zeitgeber intensities, namely *Z*_1_ = 0.05 (*blue color-map*) and *Z*_1_ = 0.1 (*red* color-map). **(B)** Entrainment phases *ψ* as a function of the photoperiod ϰ for different intrinsic periods τ, obtained from the Arnold onion for *Z*_1_ = 0.1. The curves correspond to the vertical cross-sections depicted by dashed gray lines in **(A)**. Other oscillator parameters were *A* = 1 and λ = 0.5 h^−1^.

Figure 5A shows the Arnold onions in the photoperiod (ϰ) – internal period (τ) parameter plane for two different maximal Zeitgeber intensities, namely *Z*_1_ = 0.05 and *Z*_1_ = 0.1. Please note that a right-tilted Arnold onion in the ϰ − *T* parameter plane appears as a left-tilted onion in the ϰ − *t* parameter plane and *vice versa*. Hence, Figure 5A shows an oscillator with τ_LL_ > τ_DD_ and owing to Aschoff’s rule is representative for night-active animals. Again, the amount of tilt and the entrainment region is determined by the maximal Zeitgeber intensity *Z*_1_ - the larger tilt and entrainment region is generated by a larger *Z*_1_.

In Figure 5B, we show the entrainment phase *ψ* in dependence on the photoperiod ϰ for three sections through the Arnold onion for *Z*_1_ = 0.1, which are denoted as vertical dashed lines in Figure 5A. The difference between the sections is the choice of the internal period τ. The variation in the internal periods can be interpreted as different period phenotypes of the same organism, either describing natural variation or specific clock mutants. In the first case with τ = 22 h, the vertical line crosses just one side of the onion and the entrainment phase does not span a 12 h range as discussed in Ref. (13). In the second case with τ = 26 h, the situation is mirrored: the line crosses the right border of the onion and the entrainment phase varies again within a range smaller than 12 h. With τ = 23.5 h, however, both borders of the onion can be reached by varying ϰ and the entrainment phase spans a broader range of values, appearing above as well as below the L to D transition in Figure 5. In this case, under variation of the photoperiod ϰ, the phase of entrainment spans a range considerably larger than 12 h. We additionally note that since it is the difference between the periods τ − *T* that determines the form of the Arnold onion, similar results can be obtained with a constant τ, but rather different Zeitgeber periods *T*.

## 4 Discussion

Our main finding in this manuscript is the concept of the Arnold onion, which formalizes the notion of entrainment of circadian systems under different photoperiods. Using a generic model of the circadian clock we provided theoretical evidence that the entrainment region in the photoperiod (ϰ) – Zeitgeber period (*T*) parameter plane constitutes an onion-shaped geometry, see Figure 1B. This Arnold onion has its widest entrainment range close to equinox and tapers for extreme photoperiods toward the free-running periods τ_LL_ and τ_DD_ under constant conditions. From this it follows that the Arnold onion is tilted, whenever the free-running period under a constantly *active* forcing signal (τ_LL_) is considerably different compared to its period under a constantly *inactive* forcing signal (τ_DD_), i.e., Δτ = τ_LL_ − τ_DD_ ≠ 0, see e.g., Figure 2. Owing to Aschoff’s rule, we thus expect a left-tilted Arnold onion in case of diurnal animals or plants while expecting a right-tilted Arnold onion for nocturnal animals. This proposed behavior is confirmed by a numerical study of the Arnold onions from a previously published molecular model of the plant model organism *Arabidopsis thaliana*, see Figure S5 in Supplementary Material.

A direct implication of the tilt of the Arnold onion is that the entrainment range along the photoperiodic axis is not symmetrical around equinoctial photoperiods (ϰ = 0.5). If the entraining period *T* is closer to τ_DD_ than to τ_LL_, then the oscillator will better entrain to extremely short photoperiods than to extremely long photoperiods and *vice versa* if *T* is closer to τ_LL_. This theoretical prediction is consistent with experimental findings in a set of organisms (14, 15). For example, it was found that the drinking behavior of squirrel monkeys (*Saimiri sciureus*) remained synchronized with the 24 h rhythms of light pulses even for extremely short photoperiods while the new world monkeys were not able to entrain to photoperiods longer than 21 h (i.e., LD21:3 or ϰ = 0.875) (15).

Apart from light, temperature cycles can act as Zeitgeber signals among a variety of organisms and tissues (42, 43). One of the most striking features of circadian clocks is temperature compensation, i.e., the relative independence of the circadian clocks’ free-running rhythm under constant ambient temperatures of different magnitude, at least inside the physiologically relevant range (44, 45). Since a mismatch between τ_LL_ and τ_DD_ is a necessary prerequisite for observing a tilt of the Arnold onion, such behavior is *not* expected to occur in temperature compensated circadian clocks with temperature pulses being used as an entrainment cue.

Finally, we investigated systematically how oscillator and Zeitgeber properties determine the phase of entrainment: Our results suggest an increasing phase-sensitivity on the period mismatch τ − *T* with an increasing distance from equinoctial photoperiods. It is thus expected that variations in the driving period *T* lead to larger changes of the phase of entrainment under long or short photoperiods when compared to equinox. This has been observed for experimental studies, e.g., in golden hamsters (*Mesocricetus auratus*) or fruit flies (*Drosophila pseudoobscura*) (3). Analogously, we can deduce that for a fixed Zeitgeber period *T*, small variations of the internal period τ will have an increasing impact on the resulting entrainment phase under photoperiods that increasingly differ from equinox. On the organismal level, the phase of entrainment can be associated with the chronotype of an organism (13). Thus, the distribution of different chronotypes in a population of a given species is expected to be broader under extreme photoperiods compared to equinoctial ones. On the tissue level, small variations of the intrinsic oscillator properties, e.g., of single neurons, could lead to more dramatic phase variations under extreme photoperiods when compared to equinox. Given the experimentally observed differences of the internal period across different sections in the SCN (46, 47), the higher phase-sensitivity found by our modeling approach could contribute to explain the drastic phase heterogeneity of up to 180° under extremely long photoperiods (48).

From an evolutionary point of view, the entrainment of the circadian clock by rhythmic Zeitgeber signals is of major importance. It allows an organism to phase-lock or schedule (circadian clock regulated) physiological processes around the day. Daily changes of environmental cues can thus be anticipated and optimally used. Furthermore, it opens the possibility to use the time of the day as an ecological niche (49). However, apart from adaption to daily environmental changes, organisms also adapted to seasonal changes of environmental properties. A plethora of physiological processes among a variety of organisms have been reported to react on changes in day-length, a phenomenon commonly known as *photoperiodism*: it has been shown that the photoperiod affects the growth and development of plants and triggers their onset of flowering (50, 51), triggers the induction of diapause in insects (52), and has an impact on the reproduction and the onset of hibernation in mammals (53–55). *Erwin Bünning* pioneered the proposition that it is also the circadian clock that tracks seasons by sensing the photoperiod (56). A flexible phase of entrainment might confer an advantage with respect to the adaption or evolutionary adjustment to seasonally changing demands on an organism. From the viewpoint of the flexibility of the entrainment phase, the Arnold onion offers a richer phase dynamics in comparison to the classical Arnold tongue. In the latter, the 12 h range of entrainment phase is achieved only by variations along the horizontal axis, i.e., by changing either the internal period τ or the Zeitgeber period *T*, see Ref. (16). In the case of the Arnold onion, variations both in periods τ and *T* as well as variations in the photoperiod ϰ can lead to variability of the entrainment phase even beyond the range of 12 h.

Throughout our studies, we investigated the effect of varying photoperiods among Zeitgeber signals that switch in a binary fashion between states of high (*Z*(*t*) = *Z*_1_) and low intensities (*Z*(*t*) = 0). This can only be reached under laboratory conditions. Organisms living under natural environmental conditions are usually faced with complex twilight transitions, photoperiods, and variances of light-intensity and quality due to weather. All these properties vary in a latitude- and altitude-dependent fashion while seasons pass (57). Additionally, gating phenomena on the molecular or behavioral level can influence the effective Zeitgeber strength a given organism can process. It has been shown, for example, that the light input pathway in plants is regulated by the circadian clock itself (58, 59) while sleeping behavior or burrowing can limit the amount of light received by mammals (60).

A certain amount of care should be taken with the interpretation of the dynamics on large networks of coupled oscillators: our current analysis applies to a cluster of synchronizing cells where the group behavior can be approximated by the average, i.e., when the coupling is strong, the network connections are isotropic and the population is homogeneous. Any deviation from the average behavior would imply a particular structural quality of the network. Electrophysiological (35, 61) as well as reporter gene (46, 48) data suggest a redistribution of network properties and organization under varying photoperiods in the suprachiasmatic nuclei of mice. This in turn is likely to affect the sensitivity of the whole network to Zeitgeber signals, thus potentially altering the *effective* Zeitgeber intensity. However, our current work does help set a theoretical baseline so we can pick up the unique bias in a given network. Furthermore, it still yields an attractive explanation for organismal data as given, e.g., in Ref. (3, 14, 15).

Finally, it should be noted that although light appears to be the most important Zeitgeber in most animals and “higher” plants, a variety of other signals like temperature cycles (42, 59) or odor (62) can additionally act as an entrainment cue. Since the importance of the individual Zeitgeber signals relative to each other is not known yet, an integrative view on the entrainment given all these entrainment cues at parallel remains challenging. If any of the above mentioned phenomena impacts the *effective* Zeitgeber strength in a photoperiod dependent manner, the resulting onion-shaped geometry might differ from its rather symmetric shape as depicted in Figure 1B (e.g., broader ranges of entrainment under short-day compared to long-day conditions or *vice versa*). However, an analysis of these complex yet interesting variations in the *effective* Zeitgeber signal a given organism can sense will be subject of future investigations.

## 5 Author Contributions

CS performed numerical computations and wrote text, JM designed the study, GB wrote text and designed the study, HH conceived the study.

## Conflict of Interest Statement

The authors declare that the research was conducted in the absence of any commercial or financial relationships that could be construed as a potential conflict of interest.

## Supplementary Material

The Supplementary Material for this article can be found online at http://journal.frontiersin.org/article/10.3389/fneur.2015.00094/abstract
