# Corrigendum: A Theoretical Study on Seasonality

**DOI:** 10.3389/fneur.2015.00159

**Published:** 2015-07-16

**Authors:** Christoph Schmal, Jihwan Myung, Hanspeter Herzel, Grigory Bordyugov

**Affiliations:** ^1^Institute for Theoretical Biology, Charité Universitätsmedizin, Berlin, Germany; ^2^RIKEN Brain Science Institute, Wako, Japan; ^3^Institute for Theoretical Biology, Humboldt Universität zu Berlin, Berlin, Germany

**Keywords:** circadian clock, entrainment, seasonality, oscillator, photoperiod

## *Zeitgeber* Function

Equation (15) in Section 1.3 of the Supplementary Data of (1) which originally read as

$$Z\left(t\right)=Z_{1}\left(0.5+\text{arctan}\left[S\cdot \mu \cdot \left\{\cos\left(\frac{2\;\pi \;t}{T}\right)-\cos\left(\varkappa \;\pi \right)\right\}\right]\right)$$

should be replaced by

$$Z\left(t\right)=Z_{1}\left(0.5+\frac{1}{\pi }\text{arctan}\left[S\cdot \mu \cdot \left\{\cos\left(\frac{2\;\pi \;t}{T}\right)-\cos\left(\varkappa \;\pi \right)\right\}\right]\right)\;.$$

## Funding

In addition to the funding information given in Ref. (1), we would like to acknowledge the financial support from the Strategic International Cooperative Program from the Japan Science and Technology Agency.

## Conflict of Interest Statement

The authors declare that the research was conducted in the absence of any commercial or financial relationships that could be construed as a potential conflict of interest.
