# Supplementary material for: A Theoretical Study on Seasonality
Source: Front Neurol. 2015 May 7;6:94. doi: 10.3389/fneur.2015.00094 (PMC4423511; doi:10.3389/fneur.2015.00094)
Supplement: Supplementary file 1 [file Data_Sheet_1.PDF]

Frontiers Neurology

Research Article
30 March 2015

Supplementary Material: A Theoretical Study on Seasonality

Christoph Schmal [1,*], Jihwan Myung [2], Hanspeter Herzel [3], and Grigory Bordyugov [1]

[1] *Institute for Theoretical Biology, Charité Universitätsmedizin, Philippstr. 13 Haus 4, 10115 Berlin Germany*
[2] *RIKEN Brain Science Institute, N701, Central Bldg, 2-1 Hirosawa, Wako, Saitama 351-0198, Japan*
[3] *Institute for Theoretical Biology, Humboldt Universität zu Berlin, Invalidenstraße 43 10115 Berlin Germany*

Correspondence*:
Christoph Schmal
Institute for Theoretical Biology, Charité Universitätsmedizin, Philippstr. 13 Haus 4, 10115 Berlin Germany, Christoph.Schmal@charite.de

1 SUPPLEMENTARY TEXT

1.1 OSCILLATOR MODEL

In this paper we use as an illustrative, conceptual model of the circadian clock the generic amplitude-phase-oscillator

$$\begin{aligned} \dot{r}(t) &= \lambda\, r(t)\ (A - r(t))\,, \\ \dot{\varphi}(t) &= 2\,\pi\left[\varepsilon\ \cos^2\left(\varphi(t)/2\right) + c\right] \end{aligned} \tag{1}$$

with

$$c := \sqrt{\tau^{-2} + \varepsilon^2/4} - \varepsilon/2\,, \tag{2}$$

which is a modified version of the commonly known Poincaré oscillator (**Glass and Bélair** (1986); **Glass and Sun** (1994)). It depends on a small set of only four generic parameters, namely the oscillator amplitude A, the amplitude relaxation rate λ, the intrinsic period τ, and a parameter ε controlling the phase velocity $\dot{\varphi}(t)$. The offset c was chosen such that system (1) adopts a certain intrinsic period τ for a given ε, i.e.

$$2\int_0^{\pi} \frac{\mathrm{d}\varphi}{\dot{\varphi}(t)} = \frac{1}{\sqrt{c(c+\varepsilon)}} \stackrel{!}{=} \tau\,. \tag{3}$$

When transfered into Cartesian coordinates *via* $x(t) := r(t)\,\cos(\varphi(t))$ and $y(t) := r(t)\,\sin(\varphi(t))$, Eqs. (1) can be written as

$$\begin{aligned} \dot{x}(t) &= \lambda x(t)(A - r(t)) - y(t)\left(2\pi c + \pi\varepsilon\left(\frac{x(t)}{r(t)} + 1\right)\right) =: f_x(x(t), y(t)) \\ \dot{y}(t) &= \lambda y(t)(A - r(t)) + x(t)\left(2\pi c + \pi\varepsilon\left(\frac{x(t)}{r(t)} + 1\right)\right) =: f_y(x(t), y(t))\,, \end{aligned} \tag{4}$$

where we used the abbreviation $r(t) = \sqrt{x^2(t) + y^2(t)}$.

The solutions of the dynamical equations (1) can be obtained analytically and read as

$$\begin{aligned} r(t) &= \frac{A}{e^{-\lambda A t}\left[\frac{A}{r_0} - 1\right] + 1}, \\ \varphi(t) &= 2\arctan\left[\sqrt{1+\varepsilon/c}\,\tan\left(\pi\sqrt{c(c+\varepsilon)}(t-t_0) + \arctan\left(\frac{\tan(\varphi_0/2)}{\sqrt{1+\varepsilon/c}}\right)\right)\right]. \end{aligned} \tag{5}$$

From (5) follows that $\lim_{t\to\infty} r(t) = A$ for all $r_0 \in \mathbb{R}_+ \setminus \{0\}$. In other words, all positive initial values $r_0 := \sqrt{x^2(t_0) + y^2(t_0)} \neq 0$ reach a stable limit cycle of amplitude A after some transient dynamics.

1.2 ROTATIONAL EQUIVARIANCE

In case of $\varepsilon = 0\,\mathrm{h}^{-1}$, system (1) constitutes the classical Poincaré oscillator (**Glass and Mackey**, 1988, Chapter 2.3) with uniform phase velocity

$$\dot{\varphi}(t) = 2\,\pi/\tau =: \omega\,. \tag{6}$$

In this case, system (4) simplifies to

$$\dot{\vec{x}}(t) = \begin{pmatrix} \lambda(A - r(t)) & -\omega \\ \omega & \lambda(A - r(t)) \end{pmatrix} \begin{pmatrix} x(t) \\ y(t) \end{pmatrix} =: \vec{f}(\vec{x})\,. \tag{7}$$

It can be easily shown that the right-hand-side of equation (7) yields the condition of *rotation equivariance*

$$\vec{f}(\vec{R}(\theta)\,\vec{x}(t)) = \vec{R}(\theta)\,\vec{f}(\vec{x}(t))\,, \tag{8}$$

with

$$\vec{R}(\theta) = \begin{pmatrix} \cos(\theta) & -\sin(\theta) \\ \sin(\theta) & \cos(\theta) \end{pmatrix} \tag{9}$$

being a rotation matrix.

Let $Z(t)$ be a scalar Zeitgeber function that acts as an additive component in a given direction that can be defined by a phase angle α. The corresponding dynamical equation with respect to (7) then becomes

$$\dot{\vec{x}} = \vec{f}(\vec{x}) + Z(t)\,(\cos(\alpha),\,\sin(\alpha))^T\,. \tag{10}$$

Suppose, $\vec{x}_0(t)$ is a solution of equation (10). Then it follows from rotation equivariance (8) that

$$\begin{aligned} \vec{R}(\beta)\frac{\mathrm{d}\vec{x}(t)}{\mathrm{d}t} &= \vec{R}(\beta)\vec{f}(\vec{x}(t)) + Z(t)\vec{R}(\beta)(\cos(\alpha),\,\sin(\alpha))^T \\ &\text{and consequently} \\ \Rightarrow \frac{\mathrm{d}(\vec{R}(\beta)\,\vec{x}(t))}{\mathrm{d}t} &= \vec{f}(\vec{R}(\beta)\,\vec{x}(t)) + Z(t)(\cos(\alpha+\beta),\,\sin(\alpha+\beta))^T \end{aligned} \tag{11}$$

holds true. In other words, $\vec{R}(\beta)\,\vec{x}_0(t)$ is a solution of the system where the original direction α of the Zeitgeber is shifted by a constant angle $\beta \in [0, 2\pi[$.

1.3 ZEITGEBER INPUT FUNCTION

While most animals living under natural environmental conditions are subject to alternating light-dark cycles with geographical (latitude, altitude, etc.) and seasonal dependencies of light intensity, photoperiod and twilight duration, animals held under laboratory conditions are commonly faced with Zeitgeber signals switching between "on" and "off" states in a binary fashion. Such a binary Zeitgeber can be readily written by means of

$$Z_{\sqcap}(t) := \begin{cases} Z_1 & \forall t:\ t \bmod (T) \leq \varkappa T \\ 0 & \text{elsewhere} \end{cases} \tag{12}$$

where we define the photoperiod $\varkappa := \frac{T_{Z_1}}{T} \in [0,1]$ as the duration of the phase T_{Z_1} with the high Zeitgeber signal Z_1 divided by the period T of the Zeitgeber signal.

However, in this study we use a square-wave-like Zeitgeber function with twilight for the sake of two reasons: Firstly, we can thus describe the (potentially important) characteristics of an continuous switching in Zeitgeber intensity as given under natural conditions. Secondly, as described in further detail in Section 2.2 of the main text, numerical continuation methods can only be appropriately applied to time-continuous dynamical systems without singularities in their evolution equation.

We motivate our Zeitgeber function as follows: A given sinusoidal function

$$f_1(t) = \cos(\Omega\, t) \tag{13}$$

with an oscillation frequency $\Omega = 2\pi/T$ and maxima centered at $t_{\text{max}} = n\,T$ with $n \in \mathbb{Z}$ is shifted along the ordinate by $-\cos(\varkappa\,\pi)$, i.e.

$$f_2(t) = \cos(\Omega\, t) - \cos(\varkappa\,\pi)\,, \tag{14}$$

such that the roots of $f_2(t)$ are $t_0^{(n)} = \frac{\varkappa T}{2} + n\,T$ with $n \in \mathbb{Z}$. This is equivalent to the fact that $f_2(t)$ adopts values larger than zero for the fraction $\varkappa$ and values lower than zero for the fraction $1 - \varkappa$ of time over a period T, respectively.

We can now define a square-wave-like Zeitgeber function with a non-zero twilight duration by using $f_2(t)$ as an argument in a sigmoidal function that saturates to the values 0 and Z_1 at its upper and lower boundaries, respectively. If, e.g., an *arcus tanges* is used as the sigmoidal component of such a function, the corresponding Zeitgeber signal could look like

$$Z(t) = Z_1\left(0.5 + \arctan\left[S \cdot \mu \cdot \left\{\cos\left(\frac{2\,\pi\,t}{T}\right) - \cos(\varkappa\,\pi)\right\}\right]\right)\,, \tag{15}$$

where S denotes the "steepness" around the switchpoints $t_0^{(n)}$, $\forall n \in \mathbb{Z}$, of eq. (15) and $\mu = \frac{\pi}{\Omega\,\sin(\varkappa\,\pi)}$ is chosen to equal the slope around these switch points for fixed Z_1 and S but varying Ω and $\varkappa$, i.e. it fulfills the condition $|Z'(t)|_{t=\frac{\varkappa T}{2}} \overset{!}{=} Z_1\,S$. Figure S1 depicts examples of $Z(t)$ for fixed $T = 24$h and $Z_1 = 1$ while varying the steepness S (A) or photoperiod $\varkappa$ (B). It can be shown that equation (15) converges to a regular square-wave function without twilight phases in the limit of $S \to \infty$, see also Figure S1 A for $S \gg 0$.

Finally it should be noted that any other sigmoidal function like the *logistic* or *Gudermannian* function, $l[x] := \frac{1}{1+e^{-x}}$ and $\text{gd}[x] := 2\arctan(e^x) - \pi/2$, respectively, could be used instead of the *arcus tangens* in order to construct a Zeitgeber function similar to equation (15).

1.4 HARMONIC ANALYSIS OF ASYMMETRIC SQUARE WAVE SIGNAL

It can be shown that the Fourier series

$$f_N(t) := \frac{a_0}{2} + \sum_{k=1}^{N} \left(a_k \, \cos\left(k\,\Omega\,t\right) + b_k \, \sin\left(k\,\Omega\,t\right)\right) \tag{16}$$

with

$$a_k = \frac{2}{T}\int_c^{c+T} dt\, f(t)\, \cos k\Omega t \tag{17}$$

and

$$b_k = \frac{2}{T}\int_c^{c+T} dt\, f(t)\, \sin k\Omega t\,, \tag{18}$$

using the abbreviation

$$\Omega := 2\pi/T\,, \tag{19}$$

uniformly converges everywhere for any piecewise-smooth T-periodic function $f(t) = f(t+T)$ (**Tolstov** (1962); **Forster** (2001)).

On the subset of one period T, equation (12) can be written as

$$Z(t) := Z_1\, \mathcal{I}_{[0,\varkappa T)}(t) \tag{20}$$

where $\mathcal{I}_X(t)$ is the indicator function defined as

$$\mathcal{I}_X(t) := \begin{cases} 1 & \text{if } t \in X \\ 0 & \text{otherwise}\,. \end{cases} \tag{21}$$

Thus the Fourier coefficients of the asymmetric square wave function of amplitude $Z_1/2$ are given by

$$a_k := \frac{2\,Z_1}{T}\int_0^T dt\, \mathcal{I}_{[0,\varkappa T)}(t)\, \cos\left(k\Omega t\right) \tag{22}$$

$$= \frac{2\,Z_1}{T}\int_0^{\varkappa T} dt\, \cos\left(k\Omega t\right) \tag{23}$$

$$= \frac{Z_1}{k\,\pi}\sin\left(2\,\pi\,k\,\varkappa\right) \tag{24}$$

and analogously

$$b_k := \frac{Z_1}{k\,\pi}\left(1 - \cos\left(2\,\pi\,k\,\varkappa\right)\right) \tag{25}$$

hence resulting in the partial sum

$$Z_N = \frac{Z_1}{\pi}\left\{\pi\,\varkappa + \sum_{k=1}^{N} k^{-1}\left[\sin(2\,\pi\,k\,\varkappa)\, \cos(k\,\Omega\,t) + [1-\cos(2\,\pi\,k\,\varkappa)]\,\sin(k\,\Omega\,t)\right]\right\}. \tag{26}$$

2 SUPPLEMENTARY FIGURES

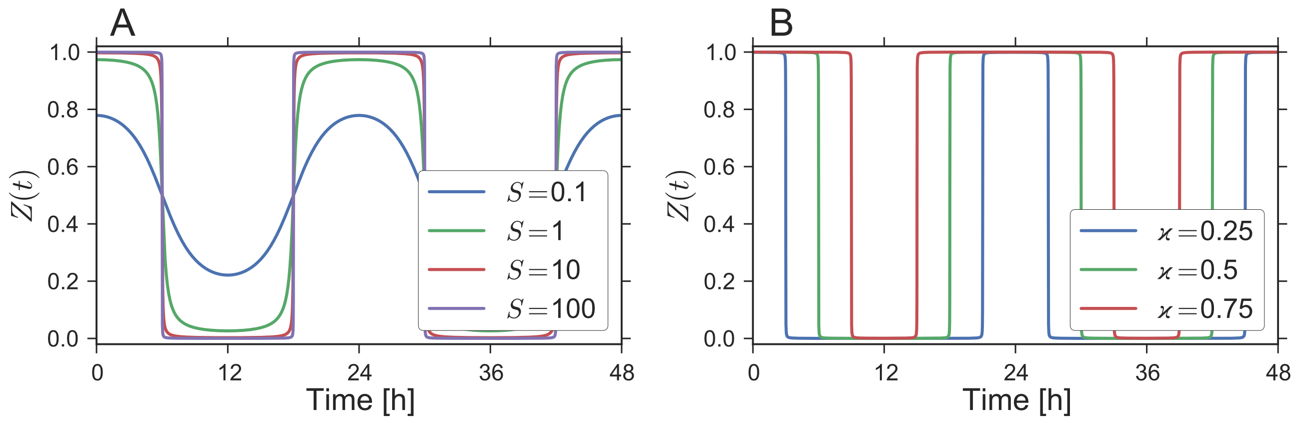

Figure S1. A) Function $Z(t)$ (see Equation (15) in Supplementary Text 1.3) is plotted versus time t for various steepnesses S but fixed $Z_1 = 1$, $T = 24$h, and $\varkappa = 0.5$. It can be noted that Equation (15) is a good approximation of the square-wave signal (12) in case of $S \gg 0$. B) Function $Z(t)$, plotted versus time t for different photoperiods $\varkappa$ but fixed $S = 100$, $Z_1 = 1$, and $T = 24$h.

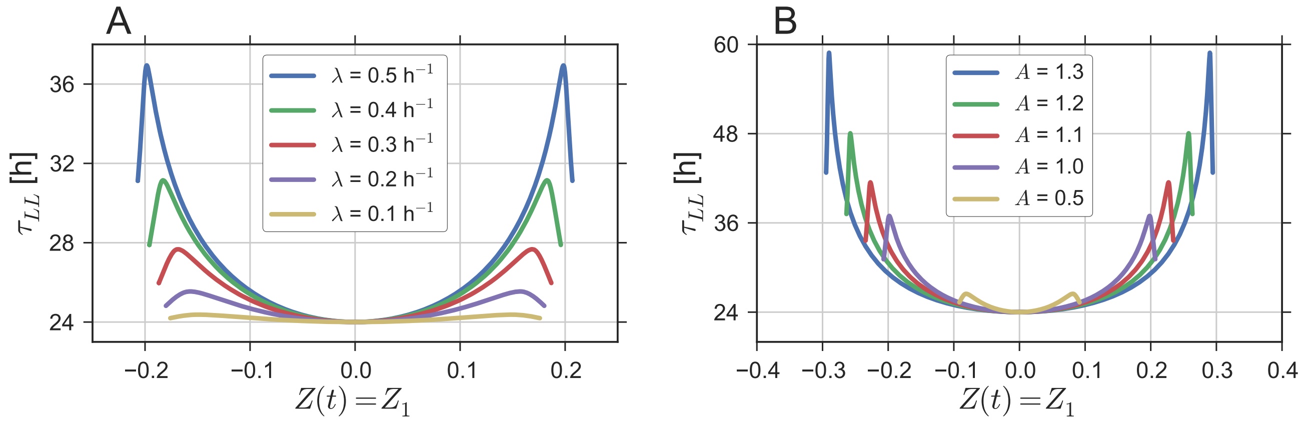

Figure S2. Free-running periods τ_{LL} as a function of the intensity Z_1 of a constant forcing signal, i.e. $Z(t) = Z_1$ for all times t, which is equivalent to setting $\varkappa$ to one in equation (12). Subplot (A) depicts the impact of different radial relaxation rates λ while fixing all other oscillator properties to $A = 1$, $\varepsilon = 0\,\mathrm{h}^{-1}$, and $\tau = 24$h. Subplot (B) depicts the impact of different oscillator amplitudes A while setting all other oscillator properties to the constant values $\lambda = 0.5\,\mathrm{h}^{-1}$, $\varepsilon = 0\,\mathrm{h}^{-1}$, and $\tau = 24$h.

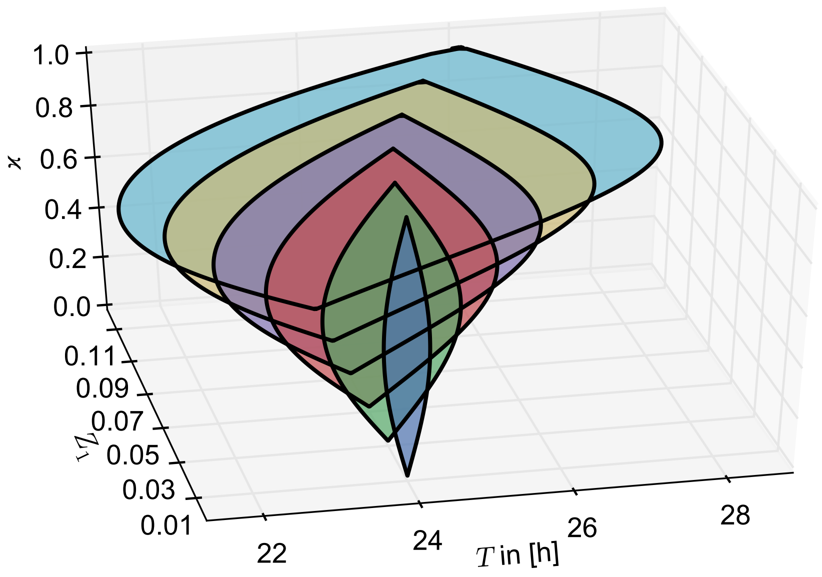

Figure S3. Three-dimensional representation of Figure 2B. Colors code for different Zeitgeber intensities *via* the same Color-Zeitgeber-Mapping as in Figure 2.

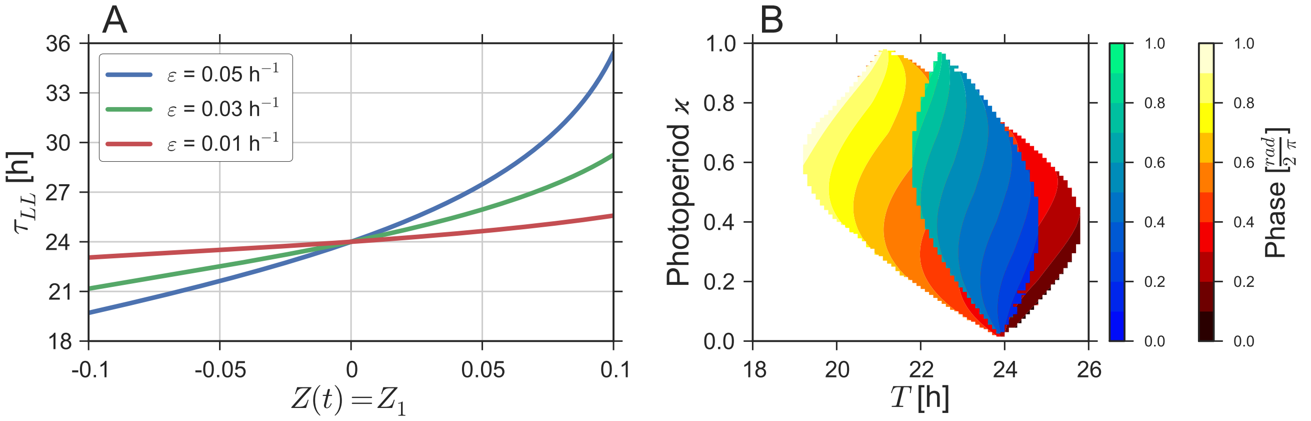

Figure S4. A) Free-running period τ_{LL} as a function of the intensity Z_1 of a constant forcing signal (i.e. $Z(t) = Z_1, \forall t$) for a *non-uniform Poincaré Oscillator* of varying ε but fixed $A = 1$, $\lambda = 0.5\,\mathrm{h}^{-1}$, and $\tau = 24$h. B) Entrainment regions in the $\varkappa - T$ parameter plane of a *non-uniform Poincaré Oscillator* with $\varepsilon = 0.03\,\mathrm{h}^{-1}$, $A = 1$, $\lambda = 0.1\,\mathrm{h}^{-1}$, and two different Zeitgeber intensities, namely $Z_1 = -0.05$ (*blue color-map*), and $Z_1 = -0.1$ (*red color-map*).

REFERENCES

Forster, O. (2001), Analysis 1, 6. Auflage (Vieweg Verlag)

Glass, L. and Bélair, J. (1986), Continuation of Arnold tongues in mathematical models of periodically forced biological oscillators, in Nonlinear Oscillations in Biology and Chemistry (Springer), 232–243

Glass, L. and Mackey, M. C. (1988), From clocks to chaos: the rhythms of life (Princeton University Press)

Glass, L. and Sun, J. (1994), Periodic forcing of a limit-cycle oscillator: Fixed points, Arnold tongues, and the global organization of bifurcations, *Physical Review E*, 50, 6, 5077

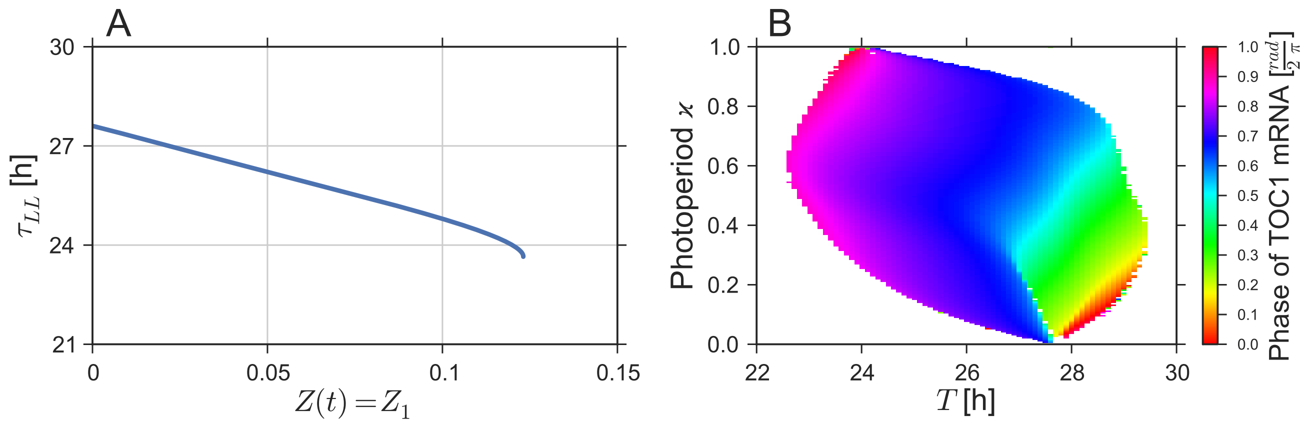

Figure S5. Free-running period τ_{LL} as a function of the intensity Z_1 (A) as well as an Arnold onion (B) for a previously published molecular model of the *Arabidopsis thaliana* circadian clock consisting of 19 differential equations and 90 model parameters (**Pokhilko et al.** (2010)). For details on the dynamical equations as well as its parameterization, we refer to the Supplementary Information of (**Pokhilko et al.** (2010)). For the sake of numerical stability in the numerical continuation of the dynamical system as plotted in (A), we approximated the original Zeitgeber function from (**Pokhilko et al.** (2010)) with the Zeitgeber model (15), using the parameters $T = 24$h and $S = 10$. Please note that the molecular model from (**Pokhilko et al.** (2010)) shows the onion shaped geometry in the $\varkappa - T$ parameter plane only for small light intensities. We therefor reduced the maximal Zeitgeber strength from its nominal value $Z_1 = 1$ in (**Pokhilko et al.** (2010)) to $Z_1 = 0.12$.

Pokhilko, A., Hodge, S. K., Stratford, K., Knox, K., Edwards, K. D., Thomson, A. W., et al. (2010), Data assimilation constrains new connections and components in a complex, eukaryotic circadian clock model, *Mol Syst Biol*, 6

Tolstov, G. P. (1962), Fourier Series (Dover Publications, New York)
